# A Review of EEG Signal Features and Their Application in Driver Drowsiness Detection Systems

**DOI:** 10.3390/s21113786

**Published:** 2021-05-30

**Authors:** Igor Stancin, Mario Cifrek, Alan Jovic

**Affiliations:** Faculty of Electrical Engineering and Computing, University of Zagreb, Unska 3, 10000 Zagreb, Croatia; igor.stancin@fer.hr (I.S.); mario.cifrek@fer.hr (M.C.)

**Keywords:** drowsiness detection, EEG features, feature extraction, machine learning, drowsiness classification, fatigue detection, deep learning

## Abstract

Detecting drowsiness in drivers, especially multi-level drowsiness, is a difficult problem that is often approached using neurophysiological signals as the basis for building a reliable system. In this context, electroencephalogram (EEG) signals are the most important source of data to achieve successful detection. In this paper, we first review EEG signal features used in the literature for a variety of tasks, then we focus on reviewing the applications of EEG features and deep learning approaches in driver drowsiness detection, and finally we discuss the open challenges and opportunities in improving driver drowsiness detection based on EEG. We show that the number of studies on driver drowsiness detection systems has increased in recent years and that future systems need to consider the wide variety of EEG signal features and deep learning approaches to increase the accuracy of detection.

## 1. Introduction

Many industries (manufacturing, logistics, transport, emergency ambulance, and similar) run their operations 24/7, meaning their workers work in shifts. Working in shifts causes misalignment with the internal biological circadian rhythm of many individuals, which can lead to sleeping disorders, drowsiness, fatigue, mood disturbances, and other long-term health problems [1,2,3,4]. Besides misalignment of the internal circadian rhythms with a work shift, sleep deprivation and prolonged physical or mental activity can also cause drowsiness [5,6,7]. Drowsiness increases the risk of accidents at the workplace [8,9,10], and it is one of the main risk factors in road and air traffic accidents per reports from NASA [11] and the US National Transportation Safety Board [12].

Drowsiness is the intermediate state between awareness and sleep [13,14,15]. Terms like tiredness and sleepiness are used as synonyms for drowsiness [16,17,18]. Some researchers also use fatigue as synonymous with drowsiness [19,20]. Definitions and differences between drowsiness and fatigue are addressed in many research papers [21,22,23]. The main difference between the two states is that short rest abates fatigue, while it aggravates drowsiness [24]. However, although the definitions are different, drowsiness and fatigue show similar behavior in terms of features measured from electroencephalogram (EEG) signal [25,26,27,28]. Because of this fact, in this review paper, we consider all the research papers whose topic was drowsiness, sleepiness, or fatigue, and we make no distinction among them.

The maximum number of hours that professional drivers are allowed to drive in a day is limited, yet drowsiness is still a major problem in traffic. A system for drowsiness detection with early warnings could address this problem. The most commonly used methods for drowsiness detection are self-assessment of drowsiness, driving events measures, eye-tracking measures, and EEG measures. Among these methods, drowsiness detection systems based on the EEG signal show the most promising results [18,29].

Brain neural network is a nonlinear dissipative system, i.e., it is a non-stationary system with a nonlinear relationship between causes and effects [30]. One way to analyze brain neural network is through feature extraction from the EEG signal. The most used techniques for feature extraction are linear, such as Fast Fourier Transform (FFT). Although it is a linear method, FFT also assumes that the amplitudes of all frequency components are constant over time, which is not the case with brain oscillations, since they are non-stationary. Because of the complexity of brain dynamics, there is a need for feature extraction methods that can properly take into account the nonlinearity and non-stationarity of brain dynamics. With an increase of computational power in recent years, many researchers work on improving the feature extraction methods, and there is a growing number of various features extracted from the EEG signal.

This paper aims to review the features extracted from the EEG signal and the applications of these features to the problem of driver drowsiness detection. We review the features since the large number of features described in the literature makes it difficult to understand their interrelationships, and also makes it difficult to choose the right ones for the given problem. To our knowledge, there is no similar review work that covers all the features discussed in this review. After the EEG features review, we continue with the review of driver drowsiness detection systems based on EEG. The main goal is to gain insight into the most commonly used EEG features and recent deep learning approaches for drowsiness detection, which would allow us to identify possibilities for further improvements of drowsiness detection systems. Finally, the main contributions of our work are the following: (1) Comprehensive review, systematization, and a brief introduction of the existing features of the EEG signal, (2) comprehensive review of the drowsiness detection systems based on the EEG signal, (3) comprehensive review of the existing similar reviews, and (4) discussion of various potential ways to improve the state of the art of drowsiness detection systems.

The paper is organized as follows: In Section 2, we present the overview of the existing review papers that are close to the topic of this paper, Section 3 provides the overview of the different features extracted from the EEG signal, Section 4 reviews the papers dealing with driver drowsiness detection systems, Section 5 provides a discussion about the features and drowsiness detection systems, and Section 6 brings the future directions of research and concludes the paper.

The search for the relevant papers included in our paper was done in the Web of Science Core Collection database. The search queries used were: (1) In Section 2.1—“{review, overview} {time, frequency, spectral, nonlinear, fractal, entropy, spatial, temporal, network, complex network} EEG features“, (2) in Section 2.2—“{review, overview} driver {drowsiness, sleepiness, fatigue} {detection, classification}”, (3) in Section 3—“<feature name> EEG feature”, (4) in Section 4—“EEG driver {‘’, deep learning, neural network} {drowsiness, sleepiness, fatigue} {detection, classification}”. Beyond the mentioned queries, when appropriate, we also reviewed the papers cited in the results obtained through the query. Additional constraints for papers in Section 4 were: (1) They had to be published in a scientific journal, (2) they had to be published in 2010 or later, 2) at least three citations per year since the paper was published, (3) papers from 2020 or 2021 were also considered with less than three citations per year, but published in Q1 journals, and (4) the number of participants in the study experiment had to be greater than 10. The goal of these constraints was to ensure that only high quality and relevant papers were included in our study.

## 2. Related Work

### 2.1. Reviews of the EEG Signal Features

Stam [30] in his seminal review paper about the nonlinear dynamical analysis of the EEG and magnetoencephalogram (MEG) signals included more than 20 nonlinear and spatiotemporal features (e.g., correlation dimension, Lyapunov exponent, phase synchronization). The theoretical background of these features and dynamical systems were also covered. The paper gave an overview of the other research works that include explanations of the features from the fields of normal resting-state EEG, sleep, epilepsy, psychiatric diseases, normal cognition, distributed cognition, and dementia. The main drawback of the paper nowadays is that it is somewhat dated (from 2005) because additional approaches have been introduced in the meantime. Ma et al. [31] reviewed the most-used fractal-based features and entropies for the EEG signal analysis, and focused on the application of these features to sleep analysis. The authors concluded that using fractal or entropy methods may facilitate automatic sleep classification. Keshmiri [32], in a recent paper, provided a review on the usage of entropy in the fields of altered state of consciousness and brain aging. The author’s work is mostly domain-specific, as it emphasizes incremental findings in each area of research rather than the specific types of entropies that were utilized in the reviewed research papers. Sun et al. [33] reviewed the complexity features in mild cognitive impairment and Alzheimer’s disease. They described the usage of five time-domain entropies, three frequency-domain entropies, and four chaos theory-based complexity measures.

Motamedi-Fakhr et al. [34], in their review paper, provided an overview of more than 15 most-used features and methods (e.g., Hjorth parameters, coherence analysis, short-time Fourier transform, wavelet transform) for human sleep analysis. The features were classified into temporal, spectral, time-frequency, and nonlinear features. Besides these features, they also reviewed the research papers about sleep stages classification. Rashid et al. [35] reviewed the current status, challenges, and possible solutions for EEG-based brain-computer interface. Within their work, they also briefly discussed the most used features for brain–computer interfaces classified into time domain, frequency domain, time-frequency domain, and spatial domain.

Bastos and Schoffelen [36] provided a tutorial review of methods for functional connectivity analysis. The authors aimed to provide an intuitive explanation of how functional connectivity measures work and highlighted five interpretational caveats: The common reference problem, the signal-to-noise ratio, the volume conduction problem, the common input problem, and the sample size problem. Kida et al. [37], in their recent review paper, provided the definition, computation, short history, and pros and cons of the connectivity and complex network analysis applied to EEG/MEG signals. The authors briefly described the recent developments in the source reconstruction algorithms essential for the source-space connectivity and network analysis.

Khosla et al. [38], in their review, covered the applications of the EEG signals based on computer-aided technologies, ranging from the diagnosis of various neurological disorders such as epilepsy, major depressive disorder, alcohol use disorder, and dementia to the monitoring of other applications such as motor imagery, identity authentication, emotion recognition, sleep stage classification, eye state detection, and drowsiness monitoring. By reviewing these EEG signal-based applications, the authors listed features observed in these papers (without explanations), publicly available databases, preprocessing methods, feature selection methods, and used classification models. For the application of drowsiness monitoring, the authors reviewed only three papers, while other applications were better covered.

Ismail and Karwowski [39] overview paper dealt with a graph theory-based modeling of functional brain connectivity based on the EEG signal in the context of neuroergonomics. The authors concluded that the graph theory measures have attracted increasing attention in recent years, with the highest frequency of publications in 2018. They reviewed 20 graph theory-based measures and stated that the clustering coefficient and characteristic path length were mostly used in this domain.

**Figure 1:**
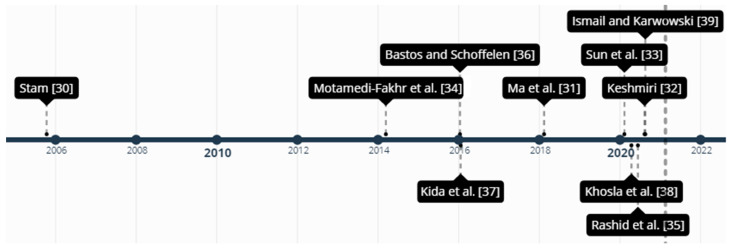
Chronologically ordered reviews of the EEG signal features.

Figure 1 shows the reviews presented in this section in chronological order of publication.

### 2.2. Reviews of the Driver Drowsiness Detection

Lal and Craig [18], in their review of driver drowsiness systems, discussed the concept of fatigue and summarized the psychophysiological representation of driver fatigue. They concluded that most studies had found a correlation of theta and delta activity with the transition to fatigue.

Lenne and Jacobs [40], in their review paper, assessed the recent developments in the detection and prediction of drowsiness-related driving events. The driving events observed were the number of line crossings, the standard deviation of lateral position, the variability of lateral position, steering wheel variability, speed adjustments, and similar events. The authors concluded that these driving performance measures correlate with drowsiness in the experimental settings, although they stipulated that the new findings from on-road studies show a different impact on performance measures. Doudou et al. [41] reviewed the vehicle-based, video-based, and physiological signals-based techniques for drowsiness detection. They also reviewed the available commercial market solutions for drowsiness detection. When it comes to the EEG signal drowsiness detection, the authors included six papers that consider frequency-domain features in this field.

Sahayadhas et al. [42] reviewed vehicle-based measures, behavior-based measures, and physiological measures for driver drowsiness detection. The section on physiological measures included 12 papers with only frequency-domain features. Sikander and Anwar [43] reviewed drowsiness detection methods and categorized them into five groups—subjective reporting, driver biological features, driver physical features, vehicular features while driving, and hybrid features. When it comes to drowsiness detection using EEG signals, the authors focused more on explaining frequency-domain features used for drowsiness detection rather than presenting research that had already been done in this field.

Chowdhury et al. [44] reviewed different physiological sensors applied to driver drowsiness detection. Observed physiological methods for measuring drowsiness included electrocardiogram (ECG), respiratory belt, EEG, electrooculogram (EOG), electromyogram (EMG), galvanic skin response (GSR), skin temperature, and hybrid techniques. Related to EEG methods, the authors included papers based on the spectral power features, event-related potentials, and entropies. The authors also discussed different materials used for dry electrodes and the problem of measurement intrusiveness for the drivers.

Balandong et al. [45] split driver drowsiness detection systems into six categories based on the used technique—(1) subjective measures, (2) vehicle-based measures, (3) driver’s behavior-based system, (4) mathematical models of sleep–wake dynamics, (5) human physiological signal-based systems, and (6) hybrid systems. The authors emphasized human physiological signal-based systems, but only the systems that rely on a limited number of EEG electrodes, as these kinds of systems are more practical for real-world applications. The authors concluded that the best results were obtained when the problem was observed as a binary classification problem and that the fusion of the EEG features with other physiological signals should lead to improved accuracy.

Other review papers of driver drowsiness systems are specialized for a certain aspect of the field, e.g., Hu and Lodewijsk [46] focused on differentiating the detection of passive fatigue, active fatigue, and sleepiness based on physiological signals, subjective assessment, driving behavior, and ocular metrics, Soares et al. [47] studied simulator experiments for drowsiness detection, Bier et al. [48] put focus on the monotony-related fatigue, and Philips et al. [49] studied operational actions (e.g., optimal staff, optimal schedule design) that reduce risk of drowsiness occurrence.

**Figure 2:**
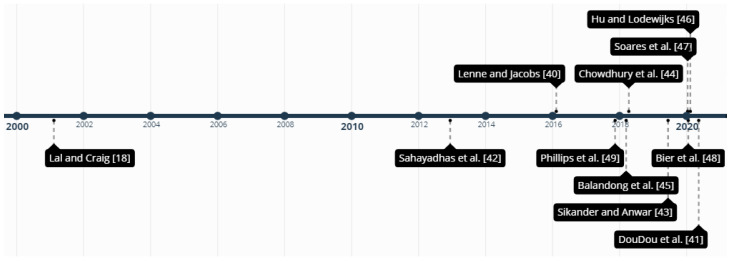
Chronologically ordered reviews of driver drowsiness detection methods.

Figure 2 shows the reviews presented in this section in chronological order of publication.

## 3. EEG Features

The purpose of this section is to introduce features that researchers extract from the EEG signal. We will not go into the details of the computation for each feature. For the readers who are interested in the detailed computation for each feature, we suggest reading the cited papers. Instead, the main idea is to present, with a brief explanation, as many features as possible, which will later allow us to identify opportunities for further improvements in the area of driver drowsiness detection. Table 1 and Table 2 show the list of all the features introduced in the following subsections. In the rest of this Section, we will use **bold** letters for the first occurrence of a particular feature name and *italic* letters for the first occurrence of a particular feature transformation or extraction method name.

### 3.1. Time, Frequency and Time-Frequency Domain Features

#### 3.1.1. Time-Domain Features

The simplest features of the EEG signal are statistical features, like **mean**, **median**, **variance**, **standard deviation**, **skewness**, **kurtosis**, and similar [50]. **Zero-crossing rate (ZCR)** [51] is not a statistical feature, yet it is also a simple feature. It is the number of times that the signal crosses the *x*-axis. The *period-amplitude analysis* is based on the analysis of the half-waves, i.e., signals between two zero-crossings. With the period amplitude analysis, one can extract the **number of waves**, **wave duration**, **peak amplitude,** and **instantaneous frequency** (IF) (based only on the single observed half-wave) [52].

**Hjorth parameters** are features that are based on the variance of the derivatives of the EEG signal. **Mobility**, **activity**, and **complexity** [53] are the first three derivatives of the signal and the most-used Hjorth parameters. Mean absolute value of mobility, activity, and complexity can also be used as a features [54]. **K-complex** [55] is a characteristic waveform of the EEG signal that occurs in stage two of the non-rapid eye movement sleep phase. **Energy** (E) of the signal is the sum of the squares of amplitude.

#### 3.1.2. Frequency-Domain Features

The power spectral density (PSD) of the signal, which is the base for calculation of the frequency domain features, can be calculated with several parametric and non-parametric methods. Non-parametric methods are used more often and include methods like Fourier transform (usually calculated with *Fast Fourier transform* algorithm, FFT [56]), *Welch’s method* [57], or *Thompson multitaper method* [58]. Examples of parametric methods for the PSD estimation are the *autoregressive* (AR) models [59], *multivariate autoregressive* models [60], or the *autoregressive-moving average* (ARMA) models [61]. The non-parametric models have a more widespread usage, because there is no need for selecting parameters such as the model’s order, which is the case for autoregressive models.

Statistical features like **mean**, **median**, **variance**, **standard deviation**, **skewness**, **kurtosis**, and similar are also used in the frequency domain. Relative powers of the certain frequency bands are the most used frequency-domain features in all fields of analysis of the EEG signals. The most commonly used frequency bands are **delta** (δ, 0.5–4 Hz), **theta** (θ, 4–8 Hz), **alpha** (α, 8–12 Hz), **beta** (β, 12–30 Hz), and **gamma** (γ, >30 Hz), band. There is also the **sigma band** (σ, 12–14 Hz) that is sometimes called **sleep spindles** [62]. Several ratios between frequency bands are widely used as features in the EEG signal analysis, i.e., **θ/α** [63], **β/α** [63], **(θ + α)/β** [64], **θ/β** [64], **(θ + α)/(α + β)** [64], **γ/δ** [65] and **(γ + β)/(δ + α)** [65].

The frequency domain of the signal can also be obtained using *wavelet decomposition* [66,67] and *matching pursuit decomposition* [68,69] methods. Unlike Fourier transform, which decomposes a signal into sinusoids, wavelet decomposition uses an underlying mother wavelet function for decomposition, and matching pursuit decomposition uses the dictionaries of signals to find the best fit for the signal.

From autoregressive models, one can extract features such as **reflection coefficients** or **partial correlation coefficients**. **Wavelet coefficients** obtained after applying wavelet decomposition can also be used as features. PSD is usually used to obtain the second-order statistics of the EEG signal. However, one can also consider the higher-order spectrum. For example, **phase coupling** [70] of different frequency components can be obtained with the higher-order spectral analysis.

#### 3.1.3. Time-Frequency Features

The analysis of the EEG signal in the domains of time and frequency simultaneously is a powerful tool, since the EEG signal is a non-stationary signal [71,72]. The most important component of time-frequency domain analysis is the possibility to observe changes in the frequency over time. Short-time Fourier transform (STFT) is the simplest function that uses uniform separation of the observed signal and calculates its frequency components. A *spectrogram* [71] can be obtained with the application of STFT. Wavelet transform [73] is the usual alternative method to spectrogram that also provides coefficients as features from the time-frequency domain. The main advantage of wavelet transform compared to spectrogram is a variable window size, dependent on spectrum frequencies.

### 3.2. Nonlinear Features

Brain dynamics constitute a complex system. A system is complex when it is constructed from many nonlinear subsystems that cannot be separated into smaller subsystems without changing their dynamical properties. Fractal systems are often used for describing the brain dynamics measured with the EEG signal. To explain fractal systems, first, we need to introduce the scaling law. The scaling law is describing (asymptomatically) a self-similar function *F* as a function of the scale parameter *s*, i.e., $F\left(s\right)\sim s^{\alpha }$. When applied to a self-affine signal, each axis should be scaled by a different power factor to obtain statistically equivalent changes in both directions. If *s* is used in the *x*-axis direction, then $s^{\prime }=s^{H}$ should be used in the *y*-axis direction. The power factor *H* is called the **Hurst exponent** [74,75]. The Hurst exponent is a measure of long-term memory of the signal and is related to the fractal dimension with the equation *D*0 = 2 − *H* for self-similar time-series, where fractal dimension *D*0 is defined in the next paragraph. Time-series *q* is monofractal if it is linearly interdependent with its **Renyi scaling exponent** τ(q), otherwise, it is multifractal. The **Renyi generalized dimension of multifractals** is defined as D(q)=τ(q)/(q−1). For more detailed explanations about fractality and multifractality of the time-series, we refer the reader to [76,77,78].

In EEG signal analysis, all fractal dimensions are estimated based on the underlying attractor (a geometric structure towards which stationary dissipative system gravitates in its state space) of the signal [79]. In a strict mathematical sense, most time-series have the one-dimensional **support fractal dimension *D*0** (or capacity dimension or Hausdorff dimension) if there are no missing values. Regardless of the value of the *D*0, the **information dimension *D*1** and **correlation dimension *D*2** [79,80,81] can be calculated. The correlational dimension *D*2 can be calculated with both monofractal and multifractal approaches. The **Katz fractal dimension** (KFD) [82], the **Petrosian fractal dimension** (PFD) [83], and the **Higuchi fractal dimension** (HFD) [84] are different approaches to the estimation of the fractal dimension. With multifractal time-series analysis, a **fractal spectrum** consisting of multiple fractal dimensions can be obtained [85,86].

Methods for fractal time-series analysis can be classified [76] into stationary analysis methods (such as *Fluctuation Analysis* [87], *Hurst’s Rescaled-Range Analysis* [74], and similar), non-stationary analysis (such as *Detrended Fluctuation Analysis* [88], *Centered Moving Average Analysis* [89], *Triangle Total Areas* [90], and similar), and multifractal analysis (such as *Wavelet Transform Modulus Maxima* [91], *Multifractal Detrended Fluctuation Analysis* [92], and similar). Each of these methods provides its own estimation of fractal dimension or scaling exponent features.

**Lyapunov exponents** (LE) [93] are measures of the attractor’s complexity. If a system has at least one positive Lyapunov exponent, then the system can be characterized as a chaotic dynamical system. A positive Lyapunov exponent points to exponential divergence of the two nearby trajectories in the attractor over time [94]. **Lempel-Ziv complexity** (LZC) [95] is a measure of complexity that binarizes time-series and then searches for the occurrence of consecutive binary characters or ‘‘words’’ and counts the number of times a new ‘‘word’’ is encountered. The **Central tendency measure** (CTM) [96] is a measure of the variability of the observed time-series and represents the percentage of points on the scatter plot that fall into a given radius. **Auto-mutual information** (AMI) [97] is a mutual information measure applied to time-delayed versions of the same EEG time-series. **Temporal irreversibility** [98] of a time-series implies the influence of nonlinear dynamics, non-Gaussian noise, or both. It is a statistical property that differs based on the direction in which time proceeds, e.g., any sequence of measurements has a different probability of occurrence than its time reverse.

A *recurrence plot* [99] is a graphical method for the detection of reoccurring patterns in the time-series. *Recurrence quantification analysis (RQA)* [100] is a group of algorithms for the automatic quantification of recurrence plots. RQA is a noise resistant method, meaning it gives good results even when the signal-to-noise ratio of considered signals is unfavorable [101]. The **recurrence rate** (RR) is the probability that a specific state of a time-series will reoccur. **Determinism** (Det) is the percentage of points that form diagonal lines on the recurrence plot and **laminarity** (Lam) is the percentage of points forming vertical lines in the recurrence plot. The **average diagonal line length** (L), **maximum length of diagonal** (Lmax), and **maximum length of vertical lines** (Vmax) are also used as RQA-based features. **Trapping time** (TT) is the average vertical line length and it relates to the predictability of the time-series. **Divergence** (Div) is the reciprocal value of the maximal diagonal line length and it can have a trend similar to the positive Lyapunov exponents. **Entropy of the recurrence plot** (ENTR) reflects the complexity of the deterministic structure of the system.

### 3.3. Entropies

Entropy was first introduced to the field of information theory by Shannon in 1948 [102,103]. **Shannon’s information entropy** is calculated based on the expression $-\underset{j}{\sum }p_{j}\log\left(p_{j}\right)$, where *p_j_* is the probability distribution of the observed data. It is used to measure uncertainty or randomness in the observed time-series. There are many derived variations of information entropy used in EEG analysis. The entropies may be considered as nonlinear features, but we describe them in a separate subsection due to their specific calculation.

**Rényi’s entropy** [104] is defined with the expression $-\frac{\alpha }{1-\alpha }\sum \log p_{k}^{\alpha },$ where α>0 and α≠1. It is a generalization of Shannon’s entropy in the case of a limited value of α→1. **Quadratic Rényi’s entropy** (or just Rényi’s entropy) is the case where α=2. **Tsallis entropy** (q-entropy) [105] is a generalization of the Boltzman–Gibbs entropy from statistical thermodynamics and is defined with the expression $\frac{k}{q-1}\left(1-\underset{i}{\sum }p_{i}^{q}\right),$ where *k* is a positive constant and *q* is the non-extensity parameter. For q>1, the entropy has a more significant reaction to the events that occur often, whereas for 0<q<1, the entropy has a more significant reaction to rare events.

The three aforementioned entropies can be calculated from the raw EEG signal. Besides that, they are a base for calculating several other entropies in the field of EEG analysis. **Kraskov entropy** (KE) [50] is an unbiased estimator of Shannon’s entropy for a *d*-dimensional random sample. **Spectral entropy** (SEN) [106] is calculated with the expression for Shannon’s entropy based on the normalized PSD of the EEG signal. **Quadratic Renyi’s spectral entropy** (QRSEN) [107] is calculated with the usage of Renyi’s entropy expression, and the difference compared to the spectral entropy is that it gives the higher weights to the lower frequencies. Commercial M-Entropy Module [108] uses two different components of spectral entropy—**response entropy** (RE) and **state entropy** (SE). State entropy includes the spectrum between 0.8 and 32 Hz, while response entropy includes the spectrum between 0.8 and 47 Hz.

**Wavelet entropy** (WE) [109,110] is somewhat similar to spectral entropy. The difference is that it is calculated based on the coefficients of the wavelet decomposition of the given time-series. There are two generalizations of wavelet entropy—**Tsallis wavelet entropy** (TWE) and **Rényi’s wavelet entropy** (RWE) [111]. **Hilbert–Huang spectral entropy** (HHSE) [112] applies Shannon’s entropy to the Hilbert–Huang spectrum, which is obtained by the *Hilbert–Huang transform* [111,113]. **Log energy entropy** (LogEn) [114] is similar to the wavelet entropy, but only uses summation of logarithms of the probabilities. **Multiresolution entropy** [115] uses the combination of windowing and wavelet transform for the detection of changes in parameters that define the observed process (i.e., the parameters of brain dynamics).

**Kolmogorov’s entropy** [116] is an embedding entropy and is defined as the sum of positive Lyapunov exponents. It represents the rate of information loss and a degree of predictability (regularity) of the attractor. Accurate computation of Kolmogorov’s entropy is computationally expensive, so several entropies are used for the estimation of Kolmogorov’s entropy based on the less computationally expensive methods. **Nonlinear forecasting entropy** [117] is the estimation of Kolmogorov’s entropy for time-series with too few points. It is based on the forecasting of the time-series data, i.e., on the correlation coefficient of the forecasted points with actually observed points. The estimation method is independent of the forecasting method used. **Maximum-likelihood entropy** [118] is also the estimation of Kolmogorov entropy. It is derived with the application of maximum-likelihood to the correlation integral, which is treated as a probability distribution. **Coarse-grained entropy** [119] is an estimation of the attractor’ entropy for cases where standardly used dimensions, Lyapunov exponents, and Kolmogorov’s entropy are not suitable due to the high dimensionality of the observed process. **Correntropy** (CoE) [120] is an estimation of nonlinear autocorrelation.

**Approximate entropy** (ApEn) [121] is derived from Kolmogorov’s entropy and its use in the analysis of the EEG signal (and other physiological signals) is widespread. It addresses the irregularity of a time-series. Predictable time-series, i.e., time-series with many repetitive patterns will have a small value of approximate entropy. **Sample entropy** (SampEn) [122] was introduced as an improvement to approximate entropy. It reduces the error of the approximate entropy by eliminating its two disadvantages—(1) self-matches and (2) dependence on the time-series length. Sample entropy is also an approximation of signal complexity. **Quadratic sample entropy** (QSE) [123] is SampEn insensitive to the data radius parameter *r*. It allows *r* to vary as needed to achieve confident estimates of the conditional probability. **Multiscale entropy** (MSE) [124] is a generalization of an entropy measure (such as sample entropy) to different time scales. **Modified multiscale entropy** (MMSE) [125] uses the same procedure as MSE, but replaces coarse-graining with a moving average procedure. **Composite multiscale entropy** (CMSE) [126] is a modification of the MSE that tackles the problem of increased variance and error estimation for short time-series.

**Permutation entropy** (PE) [127] estimates signal variability based on the repetition of the ordinal patterns. The algorithm requires parameter *m* (permutation order) to obtain ordinal patterns and their probabilities of occurrence. These probabilities are then applied in Shannon’s entropy expression. Moreover, **Renyi’s permutation entropy** (RPE) [128], **permutation Rényi entropy** (PEr) [129], **multivariate permutation entropy** (MvPE) [130], and **Tsallis permutation entropy** (TPE) [111] can be calculated for the ordinal patterns. **Dispersion entropy** (DisE) [131] is a modification of permutation entropy that tackles the problem of amplitude information loss (since permutation entropy only considers the order of the amplitude values but not the values themselves). **Amplitude-aware permutation entropy** (AAPE) [132] is based on the similar idea of using the value of the signal with the permutation entropy. **Bubble entropy** (BE) [133] is similar to permutation entropy with the main difference in the method used for ranking vectors in the embedding space. Namely, permutation entropy uses repetition of the ordinal patterns and bubble entropy uses the number of steps needed to sort a vector with the bubble sort algorithm. **Differential entropy** (DifE) [134] calculation is based on Shannon’s entropy expression and the estimation of the underlying probability density function of time-series. **Fuzzy entropy** (FuzzyEn) [135] is based on the concept of fuzzy sets, first introduced by Zadeh [136]. It is similar to sample entropy, but instead of using the Heaviside function for distance calculation, it uses a fuzzy membership function. **Transfer entropy** (TrEn) [137] uses concepts similar to mutual information (see Section 3.4) with the ability to quantify the exchange of information between two systems. It is an asymmetric measure for information transfer from process X to process Y, which measures the effect of the past values of processes X and Y on the present value of process Y.

### 3.4. Spatiotemporal Features

Features that were introduced above are all calculated based on a single EEG channel. Since EEG recording devices can have hundreds of channels nowadays, features that describe the relationship between different channels bring further insight into the understanding of brain functions. This is the main idea behind the usage of the spatiotemporal features—to describe the relationship between different brain regions for particular states or events. Spatiotemporal features can be divided into two groups—directed and non-directed. The non-directed ones relate to the synchronization of two or more channels without any knowledge of the direction, while the directed ones include the causation between them, i.e., they measure functional connectivity.

#### 3.4.1. Non-Directed Spatiotemporal Features

**Coherence** [138] is a cross-correlation equivalent in the frequency-domain, i.e., the cross-correlation of the PSD from two different channels. It reflects the synchronization of the changes of frequency components between the observed channels. **Partial coherence** [139] is an adjusted coherence with removed common signal’s linear effect based on the third channel, which is not physically close to the two observed channels. **Phase coherence** [140] is the coherence of the phases of the signals. It was introduced to overcome the problem of detection of nonlinear dependencies between the two channels.

The **phase-locking value** (PLV) [141] represents the measure of the transient phase locking that is completely independent of the signal’s amplitude, which is not the case for the coherence measure. **Coherency** [142] is calculated similar to coherence, but without applying the magnitude operator to the cross-spectral density of two channels. The obtained complex-valued quantity is called coherency. The **imaginary component of coherency** (iCoh) [143] reflects the nonlinear interaction between the two underlying time-series. **Phase-lag index** (PLI) [144] is a measure of the asymmetry of the distribution of phase differences between two signals. It brings improvement compared to the imaginary component of coherency by removing the effect of amplitude information. The **weighted phase lag index** (wPLI) [145] uses weights to reduce a phase lag index’s sensitivity to noise, while the **debiased weighted phase lag index** (dwPLI) [145] additionally reduces a sample-size bias. **Pairwise phase consistency** (PPC) [146] is a measure similar to PLV, but it quantifies the distribution of all pairwise phase differences across observations.

**Generalized synchronization** [147] incorporates the nonlinear property of the dynamical systems into its calculation. The idea is to observe two dynamical systems, a response system and a driving system, where the response system is a function of the driving system. Authors propose a numerical method called *mutual false nearest neighbors* for distinguishing between synchronized and unsynchronized behavior of the systems. *Arnhold’s measure* [148] is another algorithm for measuring such interdependence between two dynamical systems. **Synchronization likelihood** (SL) [149] brings several improvements into these methods—it is sensitive to linear and nonlinear brain dynamics and is suitable for an analysis of the non-stationary systems. It is calculated based on the similarity of the time-delayed embeddings in the state space.

**Mutual information** (MI) [150] quantifies the amount of information obtained about one time-series through observing the other time-series. It is a commonly used measure in the information theory and is calculated based on Shannon’s entropy. **Mutual information in frequency** (MIF) [151] is a recently developed measure that calculates the mutual information between the PSDs of two time-series. Its interpretation is similar to coherence.

**Cross-recurrence quantification analysis** [101] is similar to RQA, but instead of observing the self-similarity of a single signal, the similarity of two different channels is observed. The features extracted are the same as in the case of single-channel RQA (see Section 3.2). The **correlation length** (ξ_KLD_) [152] is a measure of the spatio-temporal disorder based on the Karhunen–Loeve decomposition.

#### 3.4.2. Directed Spatiotemporal Features

**Granger causality** [153] is a well-known statistical test, which tests whether one time-series forecasts (causes) the other time-series, and vice-versa. It is based on the autoregressive forecast models of the two time-series. **Spectral Granger causality** [154] can also be calculated and it is based on the estimation of the spectral transfer matrix and the covariance of the autoregressive model’s residuals. The **phase slope index** (PSI) [155] is a robust estimation of the information flow direction. It is insensitive to the mixtures of the independent sources, which is the main problem for Granger causality. Transfer entropy, which is explained in Section 3.3, can also be considered a directed spatiotemporal feature.

### 3.5. Complex Networks

The features introduced in Section 3.1, Section 3.2, and Section 3.3 were based only on a single channel of the EEG signal. Section 3.4 introduced features calculated based on the pairwise interactions between the two channels. In this section, the main goal is to introduce the features that observe the interactions between more than two channels. Complex networks are a graph-theory-based approach to EEG signal analysis. A connectivity matrix obtained by observing all pairwise connections between channels is used to obtain a graph. Any method explained in Section 3.4 can be used to determine connectivity matrix, and popular choices are correlation, PLI, or MI. Graphs can be weighted based on the values of the connectivity matrix or unweighted by applying thresholding to the connectivity matrix. A minimum spanning tree can also be used as a method for obtaining an acyclic graph with all vertices included. For more details about graph construction and complex networks, we refer the reader to papers [156,157]. In continuation of this section, we introduce features that are calculated based on the obtained graph. These features are functional connectivity features.

Once the graph is obtained, the **number of vertices** and the **number of edges** can be used as features. The **degree** (D) [158] of a vertex is the number of edges connected to the vertex. The **mean degree** of the network is a metric of density. The **degree distribution** is a probability distribution of the degrees and it provides information about the structure of the graph. **Degree correlation** (r) [159] is the correlation coefficient of degrees of pairs of neighbors in a graph. **Kappa** (k) [159] is a measure of the degree diversity and it measures the broadness of the degree distribution. The **clustering coefficient** [160] is a measure of the vertices connectedness in a graph and it can be local (for a sub-graph) or global. If the local clustering coefficient is equal to one, it means that the corresponding local sub-graph is fully connected. The global clustering coefficient is sometimes called **transitivity** [161]. A **motif** [162] is a generalized version of the clustering coefficient and a pattern of local connectivity. The average of all pairwise shortest path lengths is called **characteristic path length** [160]. **Small worldness** [163] is a second-order graph statistic and its calculation is based on the trade-off between high local clustering and short path length. **Assortativity** [164] is the measure of vertex tendency to link with other vertices with a similar number of edges.

**Efficiency** [165] is a measure of the efficiency of the information exchange in the graph. **Local efficiency** [165] is the inverse of the shortest path lengths between vertices on the observed sub-graph, where the sub-graph consists of all neighbors of the observed vertex. **Global efficiency** [165] is the average efficiency of the graph divided by the average efficiency of a fully connected graph. **Modularity** [166] describes the structure of the graph and represents the degree to which a graph is subdivided into non-overlapping clusters.

Each vertex in the graph has a measure of **centrality degree** [167], which represents the number of shortest paths in the graph that the observed vertex is involved in. Similarly, each vertex in the graph has a measure of **closeness centrality** [168], which represents the average distance of the observed vertex from all other vertices in the graph. **Eigenvalue centrality** [169] is a measure of the ease of accessibility of a vertex to other vertices. It is computed based on the relative vertex scores, with the basic idea that the high-scoring connections should contribute more to vertex influence than the low-scoring vertices. **Betweenness centrality** [170] is a measure of the importance of the vertex in a graph. It is computed based on the number of times a vertex occurs along the shortest path between two other vertices.

**Diameter** (d) [159] is the longest shortest path of a graph. **Eccentricity** (Ecc) [159] is the longest shortest path from a referenced vertex to any other vertex in the graph. **Hubs** [171] are vertices with high centrality. Hubs tend to be connected and this property is called assortativity. **Rich club** [172] is a sub-graph of highly interconnected hubs. **Leaf fraction** [159] of a graph is the number of vertices with exactly one edge. **Hierarchy** (T_H_) [159] captures the ratio between a small diameter on one hand and overloading of the hub nodes on the other hand.

## 4. Driver Drowsiness Detection Systems

The aim of this Section is to review the work on drowsiness detection focusing on the features used. The inclusion criteria for the papers are stated in Section 1. Table 3 and Table 4 show a summary of the reviewed work on driver drowsiness detection, and the rest of the Section briefly presents each work.

Balam et al. [173] used a convolutional neural network (CNN) for the classification based on the raw EEG signal from the Cz-Oz channel. They used data from the Sleep-EDF Expanded Database and their ground truth for drowsiness was the S1 sleep stage. Since the authors used a publicly available database, they compared their deep learning (DL) approach with the other feature-based approaches, and they concluded that this approach resulted in at least 3% better results. Chaabene et al. [174] used frequency-domain features for defining the ground truth. They used CNN with raw EEG signal from seven electrodes as input and achieved 90% drowsiness detection accuracy.

Yingying et al. [175] used a Long Short-Term Memory (LSTM) network to classify sleepiness in two classes and their final classification accuracy achieved was 98%. Their ground truth labels for classification were based on the alpha-blocking phenomenon and the alpha wave attenuation-disappearance phenomenon. The authors claimed that these two phenomena represent two different sleepiness levels, relaxed wakefulness and sleep onset, respectively. The authors used only the O2 channel of the EEG signal and performed a continuous wavelet transform to obtain the PSD. Zou et al. [176] used multiscale PE, multiscale SampEn, and multiscale FuzzyEn. Their ground truth labels were based on Li’s subjective fatigue scale and the accuracy achieved was 88.74%. Chaudhuri and Routray [177] used only three entropies as features—ApEn, SampEn, and modified SampEn. Their experiment was designed to slowly increase the fatigue level of the participants because of the effects of physical and mental workload, along with the effects of sleep deprivation. The experiment was divided into 11 stages and stages 7 and later were labeled as the fatigue state. The authors used SVM and achieved 86% accuracy.

Budak et al. [178] used MIT/BIH Polysomnographic EEG database in their study. Their ground truth for binary classification was based on sleep stages labeled by an expert. The awake stage was labeled the awake state and stage I of sleep was labeled the drowsy state. The authors used ZCR, E, IF, and SEN as traditional features, and also used AlexNet on the spectrogram images to obtain additional 4096 features (layers fc6 and fc7 of AlexNet). The accuracy of the binary classification was 94.31%, which is the best result achieved on this dataset, according to the authors. Mehreen et al. [179] used δ, δ/α, θ, θ/φ, δ/α+β+γ, and δ/θ EEG features, along with blink features and head movement features and achieved 92% accuracy of drowsiness detection. Based on EEG features only, the accuracy was 76%. The authors used subjective evaluation with Karolinska Sleepiness Scale (KSS) as the ground truth. It is unclear how the authors converted nine levels of KSS into a two-level ground truth. Chen et al. [180] used the clustering coefficient and characteristic path length of the graph obtained for δ, θ, α, and β frequency bands. The graph was obtained using the phase lag index. The ground truth labels were binary. The first three minutes of participants’ driving were labeled as alert state and the last three minutes as fatigue state. SVM was selected for classification and achieved 94.4% accuracy. The authors conclude that the functional connectivity of the brain differs significantly between the alert and fatigue state, particularly in the α and β bands.

Martensson et al. [181] used θ, α, θ/(θ + α), α/(θ + α), (θ + α)/β, α/β, (θ + α)/(θ + β), θ/β, SampEn, and HFD from three EEG channels together with features from EOG and ECG signals. The authors performed a sequential forward floating feature selection method for dimensionality reduction and six EEG features were selected—HFD, θ, α/(θ + α), θ/β, θ/(θ + α) and α. Random forest was selected as the best model and achieved 93.5% accuracy on the test set and 84% on the leave-one-subject-out validation scheme. The ground truth was obtained with the KSS. The severely sleepy class was for a KSS score greater than seven and the sufficiently alert class was for a KSS score of less than seven. KSS scores equal to seven were discarded as outlined. Barua et al. [182] used δ, θ, α, β, γ, (θ + α)/β, α/β, (θ + α)/(α + β), and θ/β from 30 EEG channels along with features from EOG and contextual information (e.g., time awake, duration of last sleep, and the like). The authors achieved the best accuracy of 93% for binary classification and 79% for classification into three classes. Self-evaluation with KSS score was used as ground truth and KSS score was classified into three classes—alert class for KSS scores below six, somewhat sleepy class for KSS scores below eight, and sleepy for KSS scores equal to eight or nine. In the binary classification, the authors used two methods (fuzzy centroid redistribution and SVM predicted redistribution) for redistribution of somewhat sleepy classes into the alert and sleepy classes. Ogino and Mitsukura [183] used δ, θ, α, β, and γ as frequency domain features, and parameters of the autoregressive model and MSE were also added to the feature set. Only the Fp1 channel was used and the authors achieved 67% accuracy by using SVM. The ground truth labels were based on the KSS score, where the alert class was for a KSS score less than four and the drowsy class was for a KSS score greater than six.

Chen et al. [184] analyzed the difference in complex network features for each frequency band (δ, θ, α, and β) between alert and drowsy states. The authors used the features: Number of vertices, number of edges, D, leaf fraction, d, Ecc, betweenness centrality, k, Th, and r. Their ground truth was based on the KSS score. A significant difference was found in the four features of the δ-band and five features of the θ-band. In addition, the authors suggested a more linear graph configuration in alert states and a more star-shaped graph configuration in drowsy states. Chen et al. [185] used the same experiment for drowsiness classification in a related study. Three complex network features (degree, degree correlation, and kappa) were extracted for each frequency band (δ, θ, α, and β). The ground truth was based on the KSS score and they performed binary classification. The highest accuracy of 98.6% was achieved using the *k* nearest neighbor (KNN) algorithm. Chen et al. [186] used phase synchronization, phase coherence, k, betweenness centrality, and Th as features. The first three minutes of participants’ driving were labeled as an alert state and the last three minutes as a fatigue state. The highest accuracy achieved was 95% using the extreme learning machine (ELM) algorithm. Dimitrakopoulos et al. [187] used 64 channels and computed three complex network features—clustering coefficient, characteristic path length, and small-worldness. The authors achieved 92.1% accuracy for drowsiness classification. The network values of the first and the last 5-min windows were used to indicate the states of maximum alertness and maximum fatigue, respectively.

Hong et al. [188] used δ, θ, α, β, ratio indices, frequency domain statistics, the generalized Hurst exponent, HFD, SEN, and PE from the ear channel together with photoplethysmography (PPG) and ECG. The highest accuracy achieved was 99.5%. The ground truth labels were divided into five levels and were labeled by experts based on behavioral expressions. The authors ranked the features using four different methods, and in each method, at least four of the seven best-ranked features were nonlinear features. Hu and Min [189] used 30 channels and four entropies from each channel—SEN, ApEn, SampEn, and FuzzyEn. The authors achieved 94% accuracy in drowsiness classification. They used a ground truth based on self-reported fatigue. If the measurement lasted longer than 30 min before the participant self-reported fatigue, the signals from the 5th to 10th minute were used as the normal state and the signals from the last five minutes before the end of the experiment were used as the fatigued state. Li and Chung [190] used θ, α, and β features from O1 and O2 channels along with gyroscope-based head movement measurement. The subjective Wierwille scale was used to obtain five-level ground truth. The achieved accuracy for five-level classification was 93% and for binary classification it was 96%. Awais et al. [191] used mean, variance, minimum, maximum, E, SampEn, δ, θ, α, β, and γ from 19 channels along with ECG signal. SVM was used for classification and they achieved 80% accuracy for binary classification. When only EEG features were used, the accuracy was 76%. The authors used video-based facial features including eye blink duration, facial expressions, facial tone, eye blinking rate, and movements such as head-nodding and yawning for establishing ground truth. When a drowsy event began, five minutes before it were marked as the alert state and five minutes after it were marked as the drowsy state.

Min et al. [192] used SEN, ApEn, SampEn, and FuzzyEn for fatigue detection. These four entropies gave better results than AR coefficients. An experiment was terminated based on the subjective report of fatigue. To confirm these fatigue reports, the authors utilized the Chalder Fatigue Scale and Li’s Subjective Fatigue Scale before and after the experiment. The first five minutes of the recording were labeled as the normal state and the last five minutes of the recording were labeled as the fatigued state. The authors achieved an accuracy of 98.3%. Nguyen et al. [193] used δ, θ, α, β, and γ features from 64 channels along with near-infrared spectroscopy (NIRS). EOG and ECG signals were also measured, but they were only used to establish the ground truth labels. Fisher linear discriminant analysis (FLDA) was used for binary classification with 79.2% accuracy when EEG and NIRS were used. The accuracy when only EEG features were used was 70.5%. The authors introduced the drowsiness detection index, a variable derived for drowsiness detection, and they reported that it predicts the onset of drowsiness on average 3.6 s earlier. Hu [194] used SEN, ApEn, SampEn, and FuzzyEn features from 32 channels of the EEG signal. An experiment was terminated based on the EOG parameter associated with fatigue and self-reported fatigue. The first five minutes were labeled as the normal state and the last five minutes were labeled as the fatigue state. The AdaBoost classification algorithm was used and achieved 97.5% accuracy. Chai et al. [195] used AR coefficients as features. The ground truth labels were binary, with the first five minutes of driving labeled as the alert state and the last five minutes of driving labeled as the fatigued state. An experiment was terminated when the participant drove of the road for 15 s or when consistent signs of fatigue (such as head nodding and prolonged eye closure) were detected. The authors used NN for classification and achieved 88.2% accuracy. Chai et al. [196] used AR features from 32 channels. The first five minutes of data were used as an alert state and the last five minutes as a drowsy state. The authors used a sparse deep belief network as a classification algorithm and achieved 90% accuracy. Mu et al. [197] used FuzzyEn from Fp1 and Fp2 channels and achieved 97% accuracy using the SVM algorithm. The ground truth labels were binary with the first 10 min labeled as the normal state and the last 10 min labeled as the fatigued state. The stopping criteria of the experiment were based on Li’s subjective fatigue scale and Borg’s CR-10 scale.

Fu et al. [198] used θ, α, and β features from O1 and O2 channels along with EMG and respiration. The ground truth was set based on the KSS score, where level one was KSS score equal to one or two, level two was KSS score equal to three or four, and level three was KSS score equal to five or six. The reported average area under the curve (AUC) was 0.841. When only EEG features were used, the average AUC was 0.644. Ahn et al. [199] used δ, θ, α, β, and γ along with EOG, ECG, and fNIRS. FLDA was used for binary classification with 79.2% accuracy using all the available sensors. The accuracy based only on the EEG signal features was 59.7%. Binary ground truth was used with the well-rested group and the sleep-deprived group. Huang et al. [200] used only the α feature. The system developed in this study did not use a classification algorithm. It was based on measuring the response times of the subjects. Drowsiness was labeled for the moments when the response time was 2.5 times greater than the mean response time, which helped the authors to determine a threshold for α feature value indicating drowsiness. An auditory warning system was developed to help subjects to remain alert.

Li et al. [201] used θ, α, and β features from O1 and O2 channels. The ground truth alert and drowsy data were labeled based on the percentage of eyelid closure (PERCLOS) and the number of adjustments on the steering wheel. The best accuracy of 93.16% was achieved using the SVM classifier and only θ and β features. The authors used the probability of prediction instead of the discrete class label to develop an early warning system with a probability threshold of 0.424. Chen et al. [202] used δ, θ, α, β, γ, ApEn, SampEn, Rényi’s entropy, and RQA features, along with the EOG. Two neurologists manually labeled binary ground truth values based on the EOG features and frequency domain features. ELM was used for classification based on the nonlinear features only and achieved 95.6% accuracy. Sauvet et al. [203] used θ, α, β, (θ + α)/β, and fuzzy fusion of these features. Feature thresholding was applied for classification and an accuracy of 98.3% was achieved. The ground truth was based on expert scoring, but it is unclear how this scoring was performed.

Lee et al. [204] used δ, θ, α, β, time-domain statistics, ZCR, and several ratio indices from Fpz-Cz and Pz-Oz EEG channels. The ground truth was classified into four classes: Awake, slightly drowsy, moderately drowsy, and extremely drowsy. These classes were determined by experienced physicians, with the first three classes being derived from the awake-sleep stage and the extremely drowsy class corresponding to the N1 sleep stage. SVM was used for classification and the best accuracy achieved was 98.5%. Garces Correa et al. [205] used MIT-BIH Polysomnographic Database in their research. Eighteen subjects were selected and δ, θ, α, β, γ, time-domain statistics, and frequency domain statistics features were extracted. The ground truth alert and drowsy labels were determined based on the awake and S1 sleep stages, respectively. A neural network was used for classification and it achieved 87.4% accuracy. Zhang et al. [110] used LZC and peak-to-peak versions of ApEn and SampEn. Peak-to-peak means that instead of using all the data points of the features, the authors used only the difference between the maximum and minimum values in the sliding window. Four levels of ground truth labels were used, referred to as normal state, mild fatigue, mood swing, and excessive fatigue. These labels were determined based on the various entropy patterns used in the paper, but it is unclear exactly how the labels were determined. A neural network was used for classification and it achieved 96.5% accuracy.

Hu et al. [206] used δ, θ, α, β, and frequency domain statistics along with EOG signal features. The authors achieved a final drowsiness detection accuracy of 75%. Binary ground truth labels were used. The alert state was defined with a KSS score less than 8 and Karolinska drowsiness score (KDS) equal to 0, while drowsiness was defined with a KSS score greater than 7 and a KDS score equal to or greater than 50. The KDS is an EEG/EOG-based drowsiness scoring experiment where the final score is between 0% (alert) and 100% (drowsy) [210]. Picot et al. [207] used only α and β features from the P3 channel together with the EOG signal. The ground truth was labeled by experts based on the EEG and EOG signal. Five levels were used in labeling the ground truth, but three levels were used to evaluate the drowsiness detection system. The drowsiness detection system was based on the statistical test to compare the two populations and thresholding, and achieved an accuracy of 80.6%. Zhao et al. [208] used multivariate autoregressive coefficients as features along with the EOG signal. The accuracy achieved with the SVM classifier was 81.6%. The ground truth labels were based on Li’s subjective fatigue scale. Khushaba et al. [20] introduced a hybrid type of EEG features called fuzzy mutual information-based wavelet-packet features, and achieved a drowsiness detection accuracy of 95%. Their ground truth had five levels and was based on Wierewille and Ellsworth criteria. Wierwille and Ellsworth criteria [211] is a textual description of the drowsiness continuum based on behavioral and facial signs that should prepare raters to rate participants’ drowsiness based on observations of the video while driving. Liu et al. [209] used ApEn and Kolmogorov entropy of the δ, θ, α, and β frequency bands. The ground truth was binary with pre-task time as the alert state and post-task time as the fatigue state. The authors confirmed a statistically significant increase in fatigue level based on the five different subjective scales—KSS, Stanford sleepiness scale, Samn–Perelli checklist, Li’s subjective fatigue scale, and Borg’s CR-10 scale. A hidden Markov model was used for classification and achieved 84% accuracy.

## 5. Discussion

**Figure 3:**
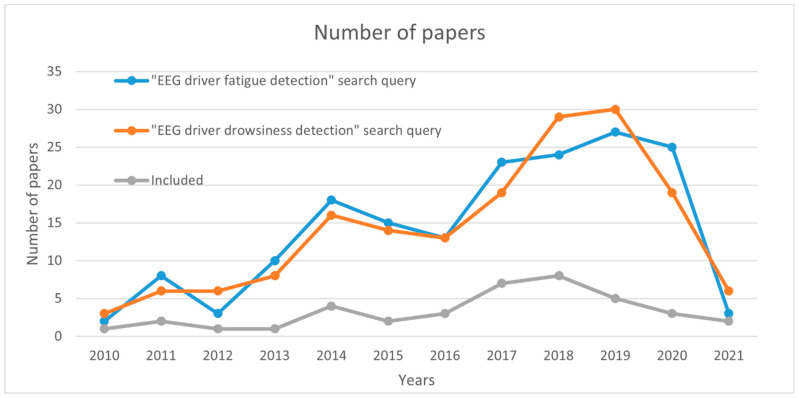
The number of papers included in the study and the number of papers obtained as a result of the “EEG driver drowsiness detection” and “EEG driver fatigue detection” search query, data until April 2021.

Section 3 presented 147 features that were classified into 7 categories, as shown in Table 1 and Table 2. As mentioned, Table 3 and Table 4 show a summary of 39 reviewed papers on drowsiness detection. The year with the most papers meeting the inclusion criteria is 2018 with eight included papers. Figure 3 shows the number of included papers and the number of papers as a result of the search query: “EEG driver drowsiness detection”. Based on both trends, it can be seen that the number of papers on this topic is increasing.

From 2013 to 2016, there were only two papers that used entropies and eight papers that used only frequency domain statistics. Although there is a higher number of published papers in recent years, there are fewer papers that rely only on frequency-domain features. Nonlinear features, entropies, and complex network features have been increasingly used in recent years. Reported drowsiness detection accuracies have remained more or less the same over the years and are usually between 80% and 99%. There is an increasing body of work that has been done with higher numbers of participants (30 or more), and it is reasonable to assume that the accuracies from these works are the most reliable.

Although we often refer to accuracy as a quality measure for the developed system, it must be noted that it is not possible to fairly compare the accuracy of different works because most of the works have been performed with a private dataset based on different experimental designs.

Besides the different datasets used, we observe that the methodology used for validation of the drowsiness detection systems is also a common problem. As mentioned earlier, EEG signal is a non-stationary and nonlinear signal with high inter-individual differences. Because of these properties, the only proper way for model validation is the validation on the signals from an unseen subject. Empirical tests show that there is a large difference in the accuracies between validation on the unseen subjects and validation on the unseen parts of the signal [212]. Reporting of validation with improper methodology can create overexpectation of the model performance, bad generalization on the unseen subjects, and can lead other researchers in the wrong direction. This effect is visible through the examples of papers that use validation on the unseen subjects, but also report about validation on the unseen parts of the signal in order to be comparable with existing research [173]. The fourth inclusion constraint defined in Section 1 was used to eliminate the papers that have a low probability of achieving good generalization due to a low number of participants.

The highest accuracy achieved was 99.5% in the work of Hong et al. [188]. It is interesting to note that the authors included features from three different categories. The authors used standard frequency bands and ratio indices, the nonlinear generalized Hurst exponent and HFD, and the entropies SEN and PE. Although this is not a large number of features, it is reasonable to assume that their diversity leads to the high accuracy of drowsiness detection. It is difficult to say how reliable the given accuracy is because only 16 participants took part in the experiment and there may be a high sampling bias in the data. The study by Martensson et al. [181] also used features from three different categories. The features used were standard frequency domain features and ratio indices, entropy SampEn, and nonlinear HFD. This study had the largest number of participants (86), and the accuracy achieved was 93.5%. These two studies suggest that using different types of features should result in high accuracy of drowsiness detection.

Complex network features for EEG signal analysis have become very popular in recent years, and this is also true for drowsiness detection systems. There are four papers [179,184,185,186] that include complex network features. One of them only provides analysis without classification and the remaining three have high accuracy—93%, 94%, and 98%. Complex networks are a promising approach, but confirming the reliability of such a system, especially when combined with features from other categories, requires studies with a large number of participants.

There is also a growing body of research on drowsiness detection using deep learning models. Deep learning models are known for their high ability to learn hidden structures in the data, but they often require a large amount of data for proper training. They can be used with the raw data as input, but also with features, or both. There are five papers using deep learning that met our inclusion criteria. In the first one, the authors used the LSTM network with raw data and different types of features and achieved 94.4% accuracy [178]. Their research was based on only 16 participants. The second one also used LSTM, but for prediction of the underlying alpha phenomena that is the base for determining drowsiness level [175]. The other three papers used CNN as a classification method. The highest accuracy achieved was 94% and the model used only raw data, without any pre-computed EEG signal features [173].

The reported accuracies for these deep learning models are in line with the accuracies of other models but, as we stated earlier, a direct comparison of the accuracies may lead to the wrong conclusions. Balam et al. [173] provided a proper comparison of different approaches. The authors used a publicly available dataset, so they were able to provide a fair comparison of different approaches. Their CNN approach was compared with one research based on the LSTM network and seven feature-based research studies. The best accuracy was obtained with their proposed method, while the LSTM method had a slightly lower accuracy. All seven feature-based approaches had more than 5% lower accuracy on average. A similar comparison was provided in Budak et al. [178] on a different publicly available dataset. Furthermore, the difference was that the authors used features and raw data for their LSTM model. The comparison was made with one deep learning approach and six other feature-based approaches. Again, the feature-based approaches had a lower performance by about 7%, on average.

These two pieces of research suggest that the deep learning approach is more appropriate and has higher performance for drowsiness detection than the feature-based approach. Nevertheless, it must be noted that all of the feature-based approaches that had lower accuracy used only time-domain and/or frequency-domain features. As shown and discussed earlier, the addition of different types of features could lead to an improvement of these models. From the inspected literature, it is currently unclear whether the inclusion of additional features would outperform deep learning models. In addition, it would be interesting to examine what effect would the addition of the features that are a measure of signal’s memory (like Hurst exponent) have, since the LSTM model also relies on the previous values of the signal. However, we can speculate that the addition of the memory-based features would increase the accuracy of these feature-based models, but probably not enough to outperform LSTM models. The reason for this is because deep models have a higher capacity for learning hidden structures than the memory-based features, but additional research should be made to support the speculation.

A larger amount of data is needed for proper training of deep learning models compared to non-deep learning models. Acquiring the data is often a problem when it comes to EEG-based drowsiness detection. Authors of research studies that use deep learning approaches often employ generative adversarial networks for the augmentation of the dataset [175]. This process often leads to an improved performance of the model. Regardless of the possibilities for augmentation of the dataset, researchers should strive to gather as much as possible real EEG signals. The larger number of participants would ensure greater diversity of the dataset, reduce the influence of inter-individual differences in EEG signals, make models more robust, and allow enough data for proper validation of models.

As we discussed earlier, there is evidence that different types of features improve drowsiness detection models. In the papers that met our inclusion criteria, about 50 different features were used, while we introduced 147 EEG-based features in our review. Approximately 100 unused features provide much room for further research. In particular, spatiotemporal features were only used to obtain a graph for complex network features [184].

Another way to improve such systems is to set better ground truth labels. Currently, many works use subjective self-evaluation as ground truth. The KSS is used most often for this purpose. The KSS is a nine-level scale, with the first four levels describing alertness, the 5th neutral level, and the last four levels describing sleepiness. The four levels for alertness and sleepiness have detailed descriptions, and they are very similar. It is also hard to tell if the scale is linear with the same distances between adjacent levels. Since it is a subjective scale with small differences between adjacent levels, it may lead to subjectivity bias and inconsistencies in the ground truth labels, which was confirmed in [191], where the authors state after the statistical test results: “Subjective measures were not reliable for detecting drowsiness alone, and that solely relying on self-reported measures may not provide a meaningful measure of a person’s actual physiological state.” Future research on how to provide a unified definition and description of drowsiness is needed to combat this subjectivity bias.

For future research, we recommend the development of a drowsiness detection system that consider raw data, features from all seven categories, and deep learning models. Ground truth labels should be based on the unified, standard definition and description of drowsiness. If there is not yet research providing such a unified definition of drowsiness, then ground truth should be confirmed with multiple independent sources to reduce subjectivity bias (even expert labels are prone to subjectivity). Because electrophysiological signals have high interindividual differences, a large number of participants (about 100 or more [181]) is needed to reduce sample bias and increase the chances of a model to have good generalization.

## 6. Conclusions

With this review paper, we bring four contributions: (1) Comprehensive review, systematization, and a brief description of the existing features of the EEG signal, (2) comprehensive review of the drowsiness detection systems, (3) comprehensive review of the existing similar reviews, and (4) discussion of various potential ways to improve the state of the art of drowsiness detection systems. In continuation, we summarize our suggestions for the general improvement of the field of drowsiness detection systems.

A higher number of participants in the experiments (about 100 or more) is needed to ensure diversity of a dataset, reduce the influence of inter-individual differences of EEG signals, make models more robust, and allow enough data for proper validation of models. Validation of EEG-based driver drowsiness detection should always be done based on the data from unseen subjects (for example, using leave-one-subject-out cross-validation). Whenever possible, datasets should be published publicly to allow fair comparison of different approaches. Based only on the papers from this review, without additional research, we were not able to identify a single feature or a feature category that guarantees the best performance of the drowsiness detection system. What we can conclude is that a higher number of features from at least four different categories should lead to more reliable drowsiness detection systems with lower sampling bias and higher generalization ability. Deep learning models exhibit higher performance for drowsiness detection than the considered non-deep learning models based on time and frequency-domain features. Nevertheless, the use of pre-computed EEG signal features together with deep learning models should always be considered (in addition to raw EEG data modeling), since in some cases, the addition of pre-computed features to deep learning models additionally boosted performance.

For future research that would have a strong impact on the field of drowsiness detection systems, we suggest the development of a unified, standard definition and description of drowsiness, which would lead to a reduction in subjective bias and easier comparison of different studies.

## Figures and Tables

**Table 1:** The list of time-domain, frequency domain and nonlinear features reviewed in this work.

Group	Feature Name	Abbr.	Group	Feature Name	Abbr.
Time-domain	Mean		Frequency-domain	θ/β	
	Median			(θ + α)/(α + β)	
	Variance			γ/δ	
	Standard deviation			(γ + β)/(δ + α)	
	Skewness			Reflection coefficients	
	Kurtosis			Partial correlation coefficient	
	Zero-crossing rate	ZCR		Wavelet coefficients	
	Number of waves			Phase coupling	
	Wave duration		Nonlinear	Hurst exponent	H
	Peak amplitude			Renyi scaling exponent	
	Instantaneous frequency	IF		Renyi gener. dim. multifractals	
	Hjorth parameters			Capacity dimension D0	D0
	Mobility			Information dimension D1	D1
	Activity			Correlation dimension D2	D2
	Complexity			Katz fractal dimension	KFD
	K-complex			Petrosian fractal dimension	PFD
	Energy	E		Higuchi fractal dimension	HFD
Frequency-domain	Mean			Fractal spectrum	
	Median			Lyapunov exponents	LE
	Variance			Lempel-Ziv complexity	LZC
	Standard deviation			Central tendency measure	CTM
	Skewness			Auto-mutual information	AMI
	Kurtosis			Temporal irreversibility	
	Delta	δ		Recurrence rate	RR
	Theta	θ		Determinism	Det
	Alpha	α		Laminarity	Lam
	Beta	β		Average diagonal line length	L
	Gamma	γ		Maximum length of diagonal	Lmax
	Sigma	σ		Max. length of vertical lines	Vmax
	θ/α			Trapping time	TT
	β/α			Divergence	Div
	(θ + α)/β			Entropy of recurrence plot	ENTR

**Table 2:** The list of entropies, undirected and directed spatiotemporal (spt.), and complex network features reviewed in this work.

Group	Feature Name	Abbr.	Group	Feature Name	Abbr.
Entropies	Shannon entropy		Undirected spt.	Imaginary component of Coh	
	Renyi’s entropy			Phase-lag index	PLI
	Tsallis entropy			Weighted phase lag index	wPLI
	Kraskov entropy	KE		Debiased weighted PLI	dwPLI
	Spectral entropy	SEN		Pairwise phase consistency	PPC
	Quadratic Renyi’s SEN	QRSEN		Generalized synchronization	
	Response entropy	RE		Synchronization likelihood	SL
	State entropy	SE		Mutual information	MI
	Wavelet entropy	WE		Mutual information in freq.	MIF
	Tsallis wavelet entropy	TWE		Cross-RQA	
	Rényi’s wavelet entropy	RWE		Correlation length	ξKLD
	Hilbert-Huang SEN	HHSE	Directed spt.	Granger causality	
	Log energy entropy	LogEn		Spectral Granger causality	
	Multiresolution entropy			Phase slope index	PSI
	Kolmogorov’s entropy		Complex networks	Number of vertices	
	Nonlinear forecasting entropy			Number of edges	
	Maximum-likelihood entropy			Degree	D
	Coarse-grained entropy			Mean degree	
	Correntropy	CoE		Degree distribution	
	Approximate entropy	ApEn		Degree correlation	r
	Sample entropy	SampEn		Kappa	k
	Quadratic sample entropy	QSE		Clustering coefficiet	
	Multiscale entropy	MSE		Transitivity	
	Modified multiscale entropy	MMSE		Motif	
	Composite multiscale entropy	CMSE		Characteristic path length	
	Permutation entropy	PE		Small worldness	
	Renyi’s permutation entropy	RPE		Assortativity	
	Permutation Rényi entropy	PEr		Efficiency	
	Multivariate PE	MvPE		Local efficiency	
	Tsallis permutation entropy	TPE		Global efficiency	
	Dispersion entropy	DisE		Modularity	
	Amplitude-aware PE	AAPE		Centrality degree	
	Bubble entropy	BE		Closesness centrality	
	Differential entropy	DifE		Eigenvalue centrality	
	Fuzzy entropy	FuzzyEn		Betweenness centrality	
	Transfer entropy	TrEn		Diameter	d
Undirected spt.	Coherence			Eccentricity	Ecc
	Partial coherence			Hubs	
	Phase coherence			Rich club	
	Phase-locking value	PLV		Leaf fraction	
	Coherency	Coh		Hierarchy	Th

**Table 3:** The summary of metadata of the reviewed driver drowsiness detection papers.

Author	Year	Participants	Electrodes
Chaabene et al. [174]	2021	12	14 channels
Balam et al. [173]	2021	23	Pz-Oz
Yingying et al. [175]	2020	12	O1 and O2
Zou et al. [176]	2020	16	32 channels
Chaudhuri and Routray [177]	2020	12	19 Channels
Budak et al. [178]	2019	16	C3-O1, C4-A1, and O2-A1
Chen et al. [179]	2019	14	14 channels
Mehreen et al. [180]	2019	50	AF7, AF8, TP9 and TP10
Martensson et al. [181]	2019	86	Fz-A1, Cz-A2 and Oz-Pz
Barua et al. [182]	2019	30	30 channels
Ogino and Mitsukura [183]	2018	29	Fp1
Chen et al. [184]	2018	15	30 channels
Chen et al. [185]	2018	15	30 channels
Chen et al. [186]	2018	12	40 channels
Hu and Min [187]	2018	22	30 channels
Dimitrakopoulos et al. [188]	2018	40	64 channels
Hong et al. [189]	2018	16	Ear channel
Li and Chung [190]	2018	17	O1 and O2
Min et al. [191]	2017	12	32 channels
Awais et al. [192]	2017	22	19 channels
Nguyen et al. [193]	2017	11	64 channels
Hu [194]	2017	28	32 channels
Chai et al. [195]	2017	43	32 channels
Chai et al. [196]	2017	43	32 channels
Mu et al. [197]	2017	11	27 channels
Fu et al. [198]	2016	12	O1 and O2
Ahn et al. [199]	2016	11	64 channels
Huang et al. [200]	2016	12	30 channels
Li et al. [201]	2015	20	O1 and O2
Chen et al. [202]	2015	16	9 channels
Sauvet et al. [203]	2014	14	C3-M2 and O1-M2
Lee et al. [204]	2014	20	Fpz-Cz and Pz-Oz
Garces Correa et al. [205]	2014	18	C3-O1, C4-A1 and O2-A1
Zhang et al. [110]	2014	20	O1 and O2
Hu et al. [206]	2013	40	Fz-A1, Cz-A2 and Oz–Pz
Picot et al. [207]	2012	20	F3, C3, P3 and O1
Zhao et al. [208]	2011	13	32 channels
Khushaba et al. [20]	2011	31	Fz, T8 and Oz
Liu et al. [209]	2010	50	13 channels

**Table 4:** The summary of reviewed driver drowsiness detection papers. The meanings of the abbreviations are: TD—time-domain, FD—frequency-domain, N—nonlinear, EN—entropies, CN—complex networks, SIG—signal-based labeling, Li’s—Li’s subjective fatigue scale, SD—sleep deprivation, NREM1—labels based on the sleep stages, BE3—first and last three minutes as two labels, BE5—first and last five minutes as two labels, BIH—behavior-based labeling, WIE—Wierwille scale, RT—reaction time based labeling, EXP—expert labeling, LSTM—long-short term memory, KNN—k nearest neighbor, SVM—support vector machine, RF—random forest, ELM—extreme learning machine, GBDT—gradient boosting decision tree, NN—neural network, FLDA—Fisher linear discriminant analysis, SDBN—sparse deep belif network, HMM—hidden Markov model, and Thres.—thresholding-based algorithm.

Author	Features	Target	Algorithm	No. Classes	Acc.
Chaabene et al. [174]	Raw	SIG	CNN	2	90.14
Balam et al. [173]	Raw	NREM1	CNN	2	94.00
Yingying et al. [175]	FD	SIG	LSTM	2	98.14
Zou et al. [176]	EN	Li’s	KNN		88.74
Chaudhuri and Routray [177]	EN	SD	SVM	2	86.00
Budak et al. [178]	TD, FD, EN and special	NREM1	LSTM	2	94.31
Chen et al. [179]	CN	BE3	SVM	2	94.40
Mehreen et al. [180]	FD	KSS	SVM	2	92.00
Martensson et al. [181]	FD, N and EN	KSS	RF	2	93.50
Barua et al. [182]	TD, FD and EN	KSS	SVM	2 and 3	93.00 and 79.00
Ogino and Mitsukura [183]	FD and EN	KSS	SVM	2	67.00
Chen et al. [184]	CN	KSS			
Chen et al. [185]	CN	KSS	KNN	2	98.60
Chen et al. [186]	CN	BE3	ELM	2	95.00
Hu and Min [187]	EN	BE5	GBDT	2	94.00
Dimitrakopoulos et al. [188]	CN	BE5	SVM	2	92.10
Hong et al. [189]	FD, N and EN	EBE	SVM	5	99.50
Li and Chung [190]	FD	WIE	SVM	5	93.87
Min et al. [191]	FD and EN	BE5	NN	2	98.30
Awais et al. [192]	TD, FD and EN	BIH	SVM	2	80.00
Nguyen et al. [193]	FD	SIG	FLDA	2	79.20
Hu [194]	EN	BE5	AdaBoost	2	97.50
Chai et al. [195]	FD	BE5	SDBN	2	90.60
Chai et al. [196]	FD	BE5	NN	2	88.20
Mu et al. [197]	EN	Li’s	SVM	2	97.00
Fu et al. [198]	FD	KSS	HMM	3	AUC 0.841
Ahn et al. [199]	FD	SD	FLDA	2	75.90
Huang et al. [200]	FD	RT			
Li et al. [201]	FD	BIH	SVM	2	93.16
Chen et al. [202]	FD, N and EN	SIG	ELM	2	95.60
Sauvet et al. [203]	FD	EXP	Threshold	2	98.30
Lee et al. [204]	TD and FD	NREM1	SVM	4	98.50
Garces Correa et al. [205]	TD and FD	NREM1	NN	2	87.40
Zhang et al. [110]	N and EN	SIG	NN	4	96.50
Hu et al. [206]	FD	KSS	SVM	2	75.00
Picot et al. [207]	FD	SIG	Threshold	5	80.60
Zhao et al. [208]	FD	Li’s	SVM	3	81.60
Khushaba et al. [20]	FD	WIE	LDA	5	95.00
Liu et al. [209]	EN	KSS and Li’s	HMM	2	84.00

## Data Availability

Not applicable.
